# SARS-CoV-2 Variant Determination Through SNP Assays in Samples From Industry Workers From Rio de Janeiro, Brazil

**DOI:** 10.3389/fmicb.2021.757783

**Published:** 2022-02-09

**Authors:** Bianca Monteiro Henriques-Santos, Bruna Farjun, Isadora Alonso Corrêa, Janaina de Barros Figueiredo, Antonio Augusto Fidalgo-Neto, Sergio Noboru Kuriyama

**Affiliations:** ^1^SESI Innovation Center for Occupational Health, Industry Federation of the State of Rio de Janeiro, Rio de Janeiro, Brazil; ^2^Department of Virology, Paulo de Góes Institute of Microbiology, Federal University of Rio de Janeiro, Rio de Janeiro, Brazil; ^3^SENAI Innovation Institute for Green Chemistry, Industry Federation of the State of Rio de Janeiro, Rio de Janeiro, Brazil

**Keywords:** SARS-CoV-2, coronavirus, variant, Alpha, Gamma, Zeta, SNP assay

## Abstract

Since the first reported case in December 2019, SARS-CoV-2 infections have become a major public health worldwide. Even with the increasing vaccination in several countries and relaxing of social distancing measures, the pandemic remains a threat especially due to the emergence of new SARS-CoV-2 variants. Despite the presence of an enzyme capable of proofreading its genome, high rates of replication provide a source of accumulation of mutations within the viral genome. In this retrospective study, samples from a cohort of industry workers tested by the SESI’s COVID-19 mass testing program from September 2020 to May 2021 were analyzed using a mutation panel in order to describe the circulation of currently identified SARS-CoV-2 variants within the samples obtained in Rio de Janeiro State. Our results demonstrated that the variant of interest (VOI) Zeta has been in circulation since October 2020 and reached 87% of prevalence in February 2021 followed by a decrease due to the emergence of Gamma variant of concern (VOC). Gamma was detected in January 2021 in our studied population, and its prevalence increased during the following months, reaching absolute prevalence within positive samples in May. The Alpha variant was detected only in 4–7% of samples during March and April while Beta VOC was not detected in our study. Our data agree with sequencing genomic surveillance databases and highlight the importance of continuous mass testing programs and variant detection in order to control viral spread and guide public health measures.

## Introduction

Mutations are an intrinsic characteristic of virus replication, especially considering RNA viruses, as is the case of SARS-CoV-2 (Almubaid and Hisham, 2021; Lauring and Emma, 2021). However, coronaviruses are less prone to mutations when compared to other RNA viruses due to an acquired enzyme capable of excising erroneous mutagenic nucleotides incorporated by their main RNA polymerase, maintaining relative integrity during replication and transcription, despite their unusually large genomes (∼30 kb) (Minskaia et al., 2006; Ferron et al., 2018). As natural selection will, in most cases, undermine the effects of arising mutations, in cases of competitive advantages regarding viral replication, transmission, or immunity escape, frequencies tend to increase. On the other hand, mutations that reduce viral fitness tend to be removed from the circulating virus community. Nevertheless, mutation frequencies are also likely to increase or decrease due to genetic drift (Lauring and Emma, 2021).

A virus with one or more new mutations is referred to as the original virus variant and can differ by one or several mutations. As mutations occur constantly, the emergence of new variants is bound to occur during a pandemic (Janik et al., 2021). Numerous SARS-CoV-2 variants have already been documented globally, all sharing one specific mutation called D614G, which became the dominant globally circulating variant after its appearance in the early COVID-19 pandemic (Huang et al., 2021). However, as the pandemic progressed, three variants of interest rapidly became predominant in several countries and have raised particular concerns: Alpha, Beta, and Gamma, which correspond to Pango lineages B.1.1.7, B.1.351, and P.1, respectively (Burki, 2021). Moreover, several variants of interest have raised awareness, among which is Zeta (Pango lineage P.2).

The SESI Innovation Center for Occupational Health (Rio de Janeiro, Brazil), of the Industry Federation of the State of Rio de Janeiro (Firjan), proposed a mass testing program for SARS-CoV-2 targeting the industry worker population of the state of Rio de Janeiro. The program focused on enabling a safe return to economic activity through early identification of infection and mitigation of virus spread in the work environment. The objective of this study was to analyze and describe the circulation of currently identified SARS-CoV-2 variants within the samples obtained in Rio de Janeiro State.

## Materials and Methods

Nasopharyngeal samples were collected from industry workers by SESI Innovation Center for Occupational Health (FIRJAN, Rio de Janeiro, Brazil)-trained nurses, as part of the COVID-19 mass testing program in the state of Rio de Janeiro. After collection, samples were maintained at 4°C in 2 ml of Dulbecco’s Modified Eagle Medium (DMEM) through initial processing.

RNA extractions were performed using Absolutely Total RNA Purification Kit (Ref. 400793, Agilent Technologies) using Agilent Technologies Bravo Automated Liquid Handling Platform, following the manufacturer’s protocols. Total RNA was stored at −80°C until further use.

After initial screening through RT-qPCR (CDC, 2020), positive samples for SARS-CoV-2 were randomly selected and a retrograde analysis was performed for mutation and variant characterization. A total of 351 samples were analyzed, spanning 45 samples from each month from November 2020 through May 2021, and 18 samples from September and October each, due to a lower positive sample pool in these 2 months. For this, we used the customizable TaqMan^®^ SARS-CoV-2 Mutation Panel SNP genotyping assay (Ref. 4332077, Applied Biosystems), and TaqPath™ 1-Step RT-qPCR Master Mix (Ref.: A15300, Applied Biosystems), following the manufacturer’s protocols for reaction volumes and cycling conditions. For targeted mutations, see Table 1. Samples identified as wild type correspond to hCoV-19/Wuhan/WIV04/2019 (accession number EPI ISL 402124).

**TABLE 1:** SNP genotyping assay targeted mutations, lineage association, gene location, and reference ID.

Mutation	Alpha (United Kingdom, B.1.1.7)	Beta (South Africa, B.1.351)	Gamma (Brazil, P.1)	Zeta (Brazil, P.2)	Gene	Assay ID
DEL69/70	X				S	AN9HXTM
N501Y	X	X	X		S	ANPRYZA
E484K		X	X	X	S	ANU7GMZ
K417N		X			S	ANZTTXP
K417T			X		S	AN49ARF
P681H	X				S	ANCFHV6

Characterization was done as follows:

• Alpha variant is characterized as positive for DEL69/70, N501Y, and P681H. Beta variant is characterized as positive for N501Y, E484K, and K417N.
• Gamma variant is characterized as positive for N501Y, E484K, and K417T. Zeta variant is characterized as positive only for E484K.
• Negative samples for all six SNP mutations were assigned as wild type.

As a positive control, previously sequenced Alpha, Gamma, and Zeta variant samples were also reanalyzed using the SNP genotyping kit for assay validation. We used one control sample for each variant lineage tested, namely, Gamma (accession number EPI_ISL_1060902; Faria et al., 2021) and Zeta (BioProject accession number PRJNA686081; Voloch et al., 2021). Alpha sequence has not been deposited yet for lack of sequencing quality.

The present study was approved by the National Committee of Research Ethics and by the Ethics Committee of Hospital Universitário Clementino Fraga Filho, protocol number 4,317,270, which waives the need for the term of informed consent.

## Results and Discussion

Rio de Janeiro has over 580,000 industry workers, from which 68,000 individuals were tested by the SESI’s COVID-19 mass testing program by the end of June 2021. Of these, none were hospitalized or showed severe symptoms at sample collection, which differentiates the presented data from clinical patient data, and is more directly extrapolatable to general population infection behavior and rates. No worker had clinical indications for testing, and tests were performed as a preventive initiative.

Due to the specific characteristics of the slice of the population reached by the program, most individuals were men, representing 72% of the samples analyzed in this study. Furthermore, selected samples for variant characterization were from individuals of legal economically active age (mainly 18–60 years old) with a Bayesian distribution (Supplementary Figure 1), mostly between 30 and 40 years old, constituting approximately 40% of total analyzed samples. The selected sample pool is representative of the total population tested, with no statistically significant difference (*p* = 0.6442, Chi-square test). Overall positive case numbers oscillated during this window, with December being the month with the highest prevalence, with a rate of 5.44%.

SARS-CoV-2 variant emergence surveillance is predominantly influenced by mutations in the spike glycoprotein, which is responsible for the binding to human cells and the major target of neutralizing antibodies. Mutations on the receptor-binding domain can modulate interactions with host angiotensin-converting enzyme II (ACE2) facilitating cell entry (Ozono et al., 2021), as is the case of D614G, present in all current variants, and N501Y, a common mutation present in variants of concern (VOCs) Alpha, Gamma, and Beta. This mutation led to lower binding free energy requirement, as well as an increased number of electrostatic interactions with ACE2 (Khan et al., 2021; Ortega et al., 2021). Spike antigenic evolution can also affect antibody immunity and vaccination efficacy since coronaviruses’ antigen modification can lead to immune system evasion (Eguia et al., 2021).

This is of special concern given that current approved vaccines tend to prevent disease instead of infection. Spreading events can alter the course of an epidemic and, therefore, prevention and detection should be prioritized. Low vaccination rates along with the drop of population adherence to sanitary measures over time, as is the case of Brazil, leads to continuous viral spread and replication, which, in turn, can also lead to new mutations and variants emergence. Novel strains can also lead to reinfection as well as new infection waves (He et al., 2021; Kirby, 2021; Resende et al., 2021). Furthermore, this could threaten the already strained healthcare resources leading to another public health emergency, requiring extended and more rigorous implementation of distancing strategies (Volz et al., 2021).

In this scenario, our results evidenced the need for constant surveillance and spread mitigation, especially in the face of new VOCs, which are characterized by increased transmissibility, virulence, aside from decreased effectiveness of public health and social measures or available diagnostics, vaccines, and therapeutics (WHO, 2021).

**FIGURE 1:**
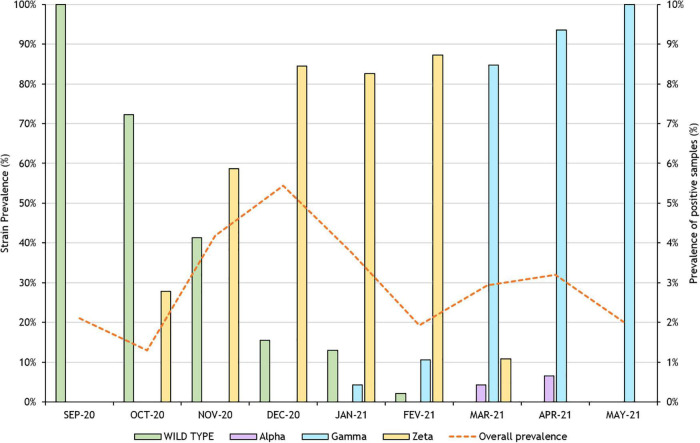
Succession pattern of variants in samples from industry workers from Rio de Janeiro in the months between September 2020 and May 2021. Analyzed variants were Alpha, Beta, and Zeta.

Considering the likeliness of the emergence of new variants and lineages carrying different mutations during virus replication, even in the presence of an error-correcting enzyme, we assessed the characterization of lineage circulation in the samples received by the program through a retrospective analysis. Validation was carried out using samples that had been previously sequenced and identified. Supplementary Tables 1, 2 show, respectively, nucleotide and amino acid sequences of the control samples. The SNP panel was able to correctly identify each lineage through the combination of specific mutations (Table 2). After initial validation, randomly selected samples were also characterized, and the resulting pattern can be seen in Figure 1.

**TABLE 2:** Variant identification in previously sequenced SARS-CoV-2 samples using SNP assay panel.

	Mutation	UK 902 #2		P1 USP		P2 814	
		Cq WILD	Cq MUT	Cq WILD	Cq MUT	Cq WILD	Cq MUT
RT-qPCR result	DEL69/70	No Cq	18.140	12.904	No Cq	24.924	No Cq
	N501Y	No Cq	18.883	No Cq	11.904	25.513	33.854
	E484K	19.773	No Cq	17.882	12.656	24.068	19.141
	K417N	20.064	No Cq	17.280	No Cq	25.834	No Cq
	K417T	20.731	27.465	23.290	12.671	25.733	33.268
	P681H	No Cq	18.950	13.039	No Cq	23.931	No Cq
Call	DEL69/70	MUTANT		WILD		WILD	
	N501Y	MUTANT		MUTANT		WILD	
	E484K	WILD		MUTANT		MUTANT	
	K417N	WILD		WILD		WILD	
	K417T	WILD		MUTANT		WILD	
	P681H	MUTANT		WILD		WILD	
	Variant	Alpha		Gamma		Zeta	

Within the sample pool analyzed in this study, the Zeta variant was firstly identified in October 2020, in one-third of positive individuals, along with a majority of samples identified as wild type. From November through February, Zeta variant predominance gradually escalated and reached 87% in the latter. Prevalence retreated to 11%, in March, with the rise of Gamma variant cases. We were unable to identify new cases of the Zeta variant by April.

Despite being firstly characterized as a new variant of interest (VOI) only in March 2021, sequencing retro analyses and genomic characterization were able to trace this lineage as far back as July 2020, in the state of Rio de Janeiro (Voloch et al., 2021). The Zeta variant continues to be considered a VOI, since its genome has mutations with established or suspected phenotypic implications, while it has been reported to cause community transmission and multiple cases (WHO, 2021). Furthermore, the Zeta variant has been associated with reinfection cases (Nonaka et al., 2021; Resende et al., 2021). This variant displays the E484K spike mutation, shared with both Beta and Gamma variants, an alteration located in the antibody binding site, directly associated with escape from neutralizing antibodies (Hoffmann et al., 2021).

Gamma variants were originally described in travelers from Amazonas, Brazil, to Japan on January 2, 2021 (Fujino et al., 2021). We were able to identify the first individuals with this variant strain by the end of January in samples from Rio de Janeiro, representing 4% of the total samples analyzed for this month. This quick spread across the country demonstrates little to no control of the virus dissemination and pandemic advance. Prevalence of Gamma variants quickly became predominant among our samples; cases jumped from 11% in February to 85% in March, reaching 100% by May. In Alagoas State, this VOC was initially diagnosed in a patient traveling from Amazonas State by mid-February, along with a case of community transmission (da Silva et al., 2021). Meanwhile, in Rio de Janeiro, there was apparently at least 13 independent events of introduction coming from nearly all regions of the country (Moreira et al., 2021b).

Meanwhile, Alpha variant’s original report dates to September 2020 and quickly became the dominant circulating SARS-CoV-2 variant in England (Public Health England [PHE], 2020). In the United States, Alpha was the dominant SARS-CoV-2 lineage, representing approximately 60% of total cases as of the beginning of May, with Delta and Gamma representing approximately 10% each (CDC, 2021). This VOC was retroactively identified in samples from October 2020 obtained from individuals from São Paulo, Brazil, and local transmission was also reported in the same federative unit, after the identification of the strain in a patient who reported no travel outside of Brazil (Claro et al., 2021; Moreira et al., 2021a). Notwithstanding, among the analyzed samples for this study, the United Kingdom variant comprised only 4 and 7% in March and April, respectively.

SARS-CoV-2 genomic data gathered by FIOCRUZ corroborates our findings, although the database comprises a different populational segment, with major sequences originating from clinical patients (Fiocruz, 2021). Prevalence profiles show great similarities in the pattern of variant succession in this time window. Considering the regionalized Southeastern compilation of lineage frequencies and the targeted variants here, the overall profile is comparable to the data produced by the present study. Additionally, April and May registered the emergence of a new variant labeled as P.4, Pango lineage, with a prevalence of 1 and 25%, respectively. This lineage has been identified in São Paulo State so far, but lacks characterization yet, and has been classified neither as a VOC nor as a VOI yet.

Although, during the pandemic, Brazil remained among the highest-ranking countries regarding the number of deaths and active cases, diagnosis for SARS-CoV-2 performed by RT-qPCR corresponded so far to a total of 55,034,721 samples, which equates to only 256,953 tests per million inhabitants (WorldOMeter, 2021). In this way, compared to other densely populated countries, Brazil continues to be one of the most affected by the virus, but also one with the fewer tests performed. Therefore, the number of cases might be highly under-reported. In fact, based on extrapolations using both serological surveys and the increase in registered deaths caused by severe acute respiratory infection, along with the increased rate of pneumonia and respiratory insufficiency in 2020, the real number of infected people could be as high as about six times greater than the number notified to the Ministry of Health (Alves et al., 2020; Hallal et al., 2020). Furthermore, as of the end of July 2021, in 7 months of the program, only approximately 19% of the total Brazilian population have been fully vaccinated, and 48% have received at least one dose of the vaccine (OurWorldinData, 2021).

## Conclusion

In conclusion, our analysis of positive samples of the 9 months between September 2020 and May 2021 describes the dynamics of the rise of two different variants among the health industry workers from Rio de Janeiro. The rapid escalation of prevalence shown demonstrates that palliation of virus dissemination is primordial to the containment of further new variants that could impair immunization and threaten healthcare. Constant vigilance through sequencing should also be prioritized, aiming to quickly identify frequent mutation in one locus, indicating the possible emergence of a lineage, along with social distancing measures. This could prevent outbreaks of new VOCs, as well as the extension of the pandemic state with the continuous emergence of waves of infected individuals.

## Data Availability Statement

The raw data supporting the conclusions of this article will be made available by the authors, without undue reservation.

## Ethics Statement

The studies involving human participants were reviewed and approved by Ethics Committee of Hospital Universitário Clementino Fraga Filho. The Ethics Committee also approved the informed consent waiver.

## Author Contributions

BH-S, BF, and SK designed and planned the study. BH-S carried out the experiments. JF prepared the samples. BH-S wrote the manuscript with support from BF, IC, and JF. AF-N helped supervise the project. SK and AF-N conceived the original idea and supervised the project. All authors contributed to the article and approved the submitted version.

## Conflict of Interest

The authors declare that the research was conducted in the absence of any commercial or financial relationships that could be construed as a potential conflict of interest. The reviewer AS declared a shared affiliation with one of the authors, IC, to the handling editor at the time of review.

## Publisher’s Note

All claims expressed in this article are solely those of the authors and do not necessarily represent those of their affiliated organizations, or those of the publisher, the editors and the reviewers. Any product that may be evaluated in this article, or claim that may be made by its manufacturer, is not guaranteed or endorsed by the publisher.
