# Supplementary material for: SARS-CoV-2 Variant Determination Through SNP Assays in Samples From Industry Workers From Rio de Janeiro, Brazil
Source: Front Microbiol. 2022 Feb 9;12:757783. doi: 10.3389/fmicb.2021.757783 (PMC8863740; doi:10.3389/fmicb.2021.757783)
Supplement: Supplementary file 1 [file Data_Sheet_1.DOCX]

Supplementary Material


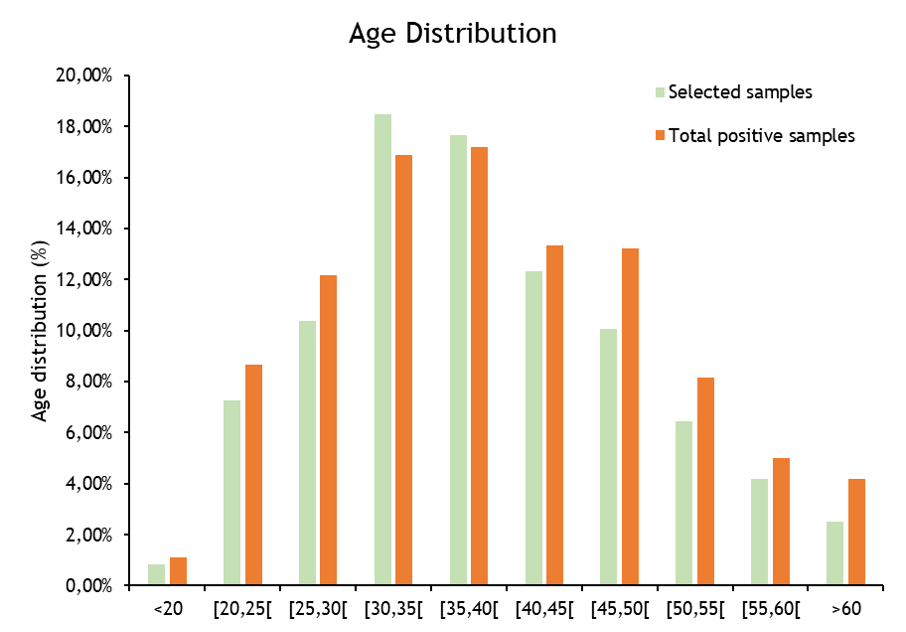


**Supplementary Figure 1.** Age distribution comparing selected samples for variant determination through SNP assay and total positive samples tested by SESI industry workers mass testing program. There are no statistically significant differences between groups. P value = 0.6442, chi-square test.
