# Supplementary material for: SARS-CoV-2 Variant Determination Through SNP Assays in Samples From Industry Workers From Rio de Janeiro, Brazil
Source: Front Microbiol. 2022 Feb 9;12:757783. doi: 10.3389/fmicb.2021.757783 (PMC8863740; doi:10.3389/fmicb.2021.757783)
Supplement: Supplementary file 2 [file Table_1.DOCX]

Supplementary Material

**Supplementary Table 1.** Nucleotide alignment of the control samples used in the study. Comparison between the wild type virus (EPI_ISL_402121), and variants Gamma (EPI_ISL_1060902, Faria et al. 2021), and Zeta (BioProject accession no. PRJNA686081, Voloch et al. 2021) showing the divergent nucleotides in the S gene of SARS-CoV-2.

| Nucleotide positions | Wild Type | Gamma | Zeta |  |
| --- | --- | --- | --- | --- |
|  |  |  |  |  |
| 21615 | C | **T** | C |  |
| 25617 | C | **T** | C |  |
| 25622 | C | **A** | C |  |
| 25639 | C | **A** | C |  |
| 25640 | C | **G** | C |  |
| 25641 | G | **C** | G |  |
| 21974 | G | **T** | G |  |
| 22130 | C | **A** | C |  |
| 22132 | T | **C** | T |  |
| 22813 | A | **C** | A |  |
| 22814 | A | **C** | A |  |
| 23012 | G | **A** | **A** |  |
| 23063 | A | **T** | A |  |
| 23065 | C | **T** | C |  |
| 23403 | A | **G** | **G** |  |
| 23404 | T | **C** | **C** |  |
| 23525 | C | **T** | **C** |  |
| 24642 | C | **T** | C |  |
| 24643 | C | **T** | C |  |
| 25088 | G | **T** | **T** |  |
| 25090 | G | **T** | **T** |  |
