# Supplementary material for: SARS-CoV-2 Variant Determination Through SNP Assays in Samples From Industry Workers From Rio de Janeiro, Brazil
Source: Front Microbiol. 2022 Feb 9;12:757783. doi: 10.3389/fmicb.2021.757783 (PMC8863740; doi:10.3389/fmicb.2021.757783)
Supplement: Supplementary file 3 [file Table_2.DOCX]

Supplementary Material

**Supplementary Table 2.** Amino acid alignment of the control samples used in the study. Comparison between the wild type virus (EPI_ISL_402121), and variants Gamma (EPI_ISL_1060902, Faria et al., 2021), and Zeta (BioProject accession no. PRJNA686081, Voloch et al., 2021) showing the divergent nucleotides in the S gene of SARS-CoV-2.

| Amino acid positions | Wild Type | Gamma | Zeta |  |
| --- | --- | --- | --- | --- |
|  |  |  |  |  |
| 18 | L | **F** | L |  |
| 20 | T | **N** | T |  |
| 26 | P | **S** | P |  |
| 138 | D | **Y** | D |  |
| 190 | R | **S** | R |  |
| 417 | K | **T** | K |  |
| 484 | E | **K** | **K** |  |
| 501 | N | **Y** | N |  |
| 614 | G | **G** | G |  |
| 655 | H | **Y** | H |  |
| 1027 | T | **I** | T |  |
| 1176 | V | **F** | **F** |  |
